# Safe Decision Controller for Autonomous DrivingBased on Deep Reinforcement Learning inNondeterministic Environment

**DOI:** 10.3390/s23031198

**Published:** 2023-01-20

**Authors:** Hongyi Chen, Yu Zhang, Uzair Aslam Bhatti, Mengxing Huang

**Affiliations:** 1 School of Computer Science and Technology, Hainan University, Haikou 570228, China; 2 School of Information and Communication Engineering, Hainan University, Haikou 570228, China

**Keywords:** autonomous driving, formal specification, deep reinforcement learning, safe decision controller generation, nondeterministic environment

## Abstract

Autonomous driving systems are crucial complicated cyber–physical systems that combine physical environment awareness with cognitive computing. Deep reinforcement learning is currently commonly used in the decision-making of such systems. However, black-box-based deep reinforcement learning systems do not guarantee system safety and the interpretability of the reward-function settings in the face of complex environments and the influence of uncontrolled uncertainties. Therefore, a formal security reinforcement learning method is proposed. First, we propose an environmental modeling approach based on the influence of nondeterministic environmental factors, which enables the precise quantification of environmental issues. Second, we use the environment model to formalize the reward machine’s structure, which is used to guide the reward-function setting in reinforcement learning. Third, we generate a control barrier function to ensure a safer state behavior policy for reinforcement learning. Finally, we verify the method’s effectiveness in intelligent driving using overtaking and lane-changing scenarios.

## 1. Introduction

Autonomous driving systems are complicated cyber–physical systems (CPS) that integrate physical environment awareness with intelligent computing, and their functioning includes both computational and physical processes that interact with and influence one another. A typical autonomous driving system includes the physical environment, plant, sensor, decision controller, and actuator. The decision controller is based on sensing physical environment information and integrating the physical environment and self-vehicle information to generate safe and reasonable driving behavior and control the vehicle’s movement.

Currently, a data-driven approach is common when obtaining autonomous driving decisions. Among data-driven approaches, training through deep reinforcement learning (DRL) is popular. Deep reinforcement learning seeks the best strategies through extensive learning training by interacting with the environment; however, for complex driverless traffic scenarios, the decision-making system cannot understand the complex traffic environment around it. Safe decision-making behavior cannot be provided for unrecognized states; therefore, the safety of deep reinforcement learning has been widely discussed.

However, most DRL tasks not only require a considerable amount of training time and iterations, but they also do not guarantee the safety of the actions taken during the learning process, which can be a serious problem in some safety-critical areas (e.g., autonomous driving, medical, etc.).

There are two main reasons why reinforcement learning is currently primarily applied in simulation environments rather than real-world scenarios. On the one hand, there is a lack of interpretability for deploying reward functions for deep reinforcement learning because the state and action space inputs for reinforcement learning come from a network of black boxes. The reward function allows the combination of prior knowledge [1]; however, reward functions based on prior human knowledge are typically subjective and sparse and thus cannot accurately represent the task goal [2]. Subjectivity, in other words, leads to reward functions that may focus on specific explicit criteria while ignoring the relationship between specific states and task goals. This lack of interpretability has become one of the main obstacles in the field. On the other hand, the real-world environment is characterized by many uncertain environmental factors. Because of the complexity of the physical environment in which autonomous driving exists, it is possible to ignore some nondeterministic environmental factors, which means that performing the same action in the same state may result in a different next state or reward value, implying that the intelligent driving system is unpredictable. This means that the autonomous driving system cannot complete the task based on the original planning decision, and it must adjust the original decision based on the cognitive outcomes of the nondeterministic environment. In the face of these uncontrolled changes in conditions, reinforcement learning cannot provide strict safety constraints for safety-critical unmanned systems.

To address the above issues, we combine formal methods and DRL to propose a DRL-based approach for generating decision controllers for autonomous driving CPS in a nondeterministic environment, where the learning process is guided by expected behavioral constraints.

First, we modeled the environment for describing the physical environment of the autonomous driving system and quantitatively evaluated its impact on the system. Second, we designed the structure of a reward machine through a nondeterministic environment model. The reward machine, based on the policy model, guides the design of the reward function, which allows the system state and goal to be combined and provides an interpretable method for the reward function. Simultaneously, we used safety properties to generate control barrier functions to monitor the safety of the decision’s output to the actuators and to improve the satisfaction of safety specifications to compensate for the DRL. Finally, we evaluated the decision controller’s performance in the overtaking lane-change scenario in terms of safety, efficiency, and satisfaction with the constraints. The following are our main research contributions:

- We proposed a method for modeling the environment of autonomous driving systems in nondeterministic environments. We quantitatively described the surrounding environmental risk by means of a well-defined nondeterministic physical environment model.
- Our proposed method combined safety verification and DRL. We defined a form of reward-machine ground description based on the environmental risk model to guide the setting of the reward function in DRL, making the reward function for DRL interpretable. We also designed a monitor using safety properties to ensure that decisions satisfying the safety properties were output to the controller.

The rest of our paper is structured as follows: Section 2 provides a synopsis of related work. Section 3 introduces the fundamental theory of metric temporal logic and reinforcement learning. Section 4 defines the general structural framework. Section 5 describes methods for modeling systems in nondeterministic environments. Section 6 proposes a decision-making generation method for safety-constrained guidance. Section 7 includes experiments and evaluations of the proposed method’s effectiveness. Finally, we draw our conclusions in Section 8.

## 2. Related Work

The safety control of a CPS system is a new research field, in which the use of safety reinforcement learning (RL) is a considerable topic. There are many methods of safety RL, which were summarized by Garcia and Fernández [3]. They divided these methods into two categories: modifying the optimality criteria and changing the learning process of agents.

Many approaches to safety RL use a restricted optimality criterion in RL. The agent searches the entire policy space for a priori safe control behaviors [4]. Xu and Mannor modified the optimality criterion to reflect the safety problem [5], providing the agent with some preliminary knowledge to guide policy exploration away from unsafe states.

It is critical to distinguish between optimization within known safety policies and an exploration of existing control options. Katz et al. [6] examined learning-process policies, which are appropriate when the learning phase is not safety critical but not when the system must operate safely while learning. Value consistency and safety policy validation are two considerable evaluation metrics in safety RL. The goal of value consistency is to ensure that the learning system’s goals are consistent with human intentions [7]. This is because RL agents can profit from the wrong goals.

The goal of policy validation is to understand how and why learned policies make certain decisions. This is critical for the secure deployment of RL agents. The task’s goal can be expressed in a reward-based or -free manner through value consistency. Reward shaping is a popular method for developing intense reward functions.

On the one hand, forming a dense reward function that is well-aligned with the true task goal can be difficult for complex tasks. Learning from demonstrations, on the other hand, allows agents to learn without an explicit reward function; to this end, J. Lee et al. [8] provided a recent overview of recent advances in value alignment. Recent value alignment efforts include iterative scaling [9,10], in which agents and personnel collaborate to learn the desired strategy (possibly during iteration). However, the approaches described above do not allow strategies to avoid risk simply by using maximizing returns as an optimization criterion.

For RL in an uncertain environment, G. Mason et al. [11] used a probabilistic model to check the verification strategy according to the constraints specified in probabilistic temporal logic; then, they modeled the environment as an abstract Markov decision process to validate the policy. However, few studies consider the worst-case scenario under uncertainty and minimax criteria, outputting a control strategy after optimizing the target to maximize the reward.

At the same time as maximizing the income expectation, Richa et al. [12] proposed that other types of utility, including risk, should be within certain given boundaries, taking such constraint-system states as fence functions and strategy space constraints or boundaries. Further research [13] examined the risk-related prior information for agents, which allows to avoid risk states during training and ensures a more efficient learning.

Another study [14] combined interpretable rule-based policies and black-box reinforcement learning to achieve system safety and robustness. Krasowski [15] used an ensemble-based approach to enhance the safety of reinforcement learning by extending the reinforcement-learning safety layer to limit the action space. Wachi et al. [16] proposed a reinforcement learning method with a constrained Markov decision process, which continuously learned security constraints by extending the security region. Bastani et al. [17] proposed a method for learning and verifying decision tree strategies. In the DRL field, strategy verification to a large extent belongs to the category of neural network verification. Strategy verification is usually used to explain the learned strategies after training. Some studies [18,19] combine temporal logic and automata to solve the non-Markov reward decision process, while other studies [20,21] use the robustness of signal sequential logic to stimulate the learning process. Wen et al. [22] combined the maximum likelihood IRL with a task constraint in the form of common safety linear sequential logic. Alshiekh et al. [23] used finite-state automata as a safety measure to supervise the agent-release operation. In our study, we used temporal logic and the corresponding automata to represent task goals and guide learning, respectively. In addition to verifying the decision, there are also interventions to ensure the safety of the training process. Hahn et al. [24] proposed a technique to compile w-regular attributes into limit-deterministic Büchi automata, which led to w-regular rewards that RL agents could learn. Toro et al. [25] introduced the reward machine, which closely reorganizes an automaton, and demonstrated its use on the RL benchmark. Araki et al. [26] developed a logic-based value iteration network, which integrated FSA into value iteration through demonstration.

Li [27] proposed a deep reinforcement learning algorithm that incorporated a risk-assessment function to find the optimal driving strategy and minimize the desired risk. However, the authors only assessed driving risk in terms of positional uncertainty and distance-based safety measures, which lacked any consideration of uncertain environmental factors, such as driving style. As such, we used a formal approach to define driving risk for nondeterministic environmental factors. Muzahid et al. [28] made several contributions to the scheme for adapting the reward function. They used a multiobjective reward system to balance achievement rewards from cooperative and competitive approaches, accident severity, and passenger comfort. However, they considered this from the perspective of multivehicle crashes and overlooked the effects of differences in speed and driving style.

In general, the lack of interpretability and ensuring security in complex environments have become considerable research topics for deep reinforcement learning research.

## 3. Basic Theory

### 3.1. Metric Temporal Logic

Linear temporal logic (LTL) is a kind of temporal logic. It models time as a series of states and describes the constraints that the system needs to meet in the past, present, and future through time operators. Linear temporal logic is used to represent the properties or constraints that the system is expected to meet. If the AP is an atomic proposition set, the LTL formula on the set AP can be recursively defined as follows [29].

(1) $$\begin{array}{c} \varphi ::=p|\neg \varphi |\varphi_{1}\wedge \varphi_{2}|\varphi_{1}\to \varphi_{2}|\varphi_{1}↔\varphi_{2}\left|G\varphi |F\varphi |X\varphi \right|\varphi_{1}U\varphi_{2} \end{array}$$

In the above definition, they are all LTL formulas, and the standard operators logical and (∧), logical or (∨), not (¬) and implication (→) are propositional logic symbols. Operators *F* (future), *G* (globally), *X* (next) and *U* (until) are sequential operators. The explanation is as follows.

- $\varphi ,\varphi_{1},\varphi_{2}$ are logical propositions, and the return value is true or false.
- G(φ):φ is always true on the time axis.
- F(φ):φ is always true on the time axis and will be true at some time in the future.
- X(φ):φ is true at the next point in time.
- $\varphi_{1}U\varphi_{2}$: Before $\varphi_{2}$ becomes true at some point in the future, $\varphi_{1}$ is continuously true.

Metric temporal logic (MTL) [30] is an extension of LTL that uses discrete-time, interval-time arithmetic to specify the time limits that must be maintained for certain temporal properties and is thus well-suited to represent real-time monitoring requirements. It is a timing logic that operates in real time. Each temporal logic operator, such as $G\left[i,j\right],F\left[i,j\right],\varphi_{1}U\left[i,j\right]\varphi_{2}$ and so on, has a subscript representing the duration of the operator. G[1,2]φ indicates that φ must be true between the times 1 and 2. LTL formulas are analogous to MTL formulas without time intervals.

### 3.2. Reinforcement Learning (RL)

RL is a method for maximizing long-term rewards in Markov decision processes (MDPs) that has found widespread application in artificial intelligence [31]. In RL training, the agent without prior knowledge must obtain the information about the environment through continuous exploration (i.e., interaction with the environment) and obtain the optimal decision strategy through repeated experiments. The agent can learn the decision-making strategies for specific tasks independently, without the need for designers to describe the implementation of tasks in detail. In the learning process of the agent, after each agent makes a tentative decision, the environment will change its state correspondingly, and give the agent a reward value (or reward) according to the changed state. According to the continuous observation of the environment and the feedback obtained, the agent will optimize its strategy in the process of continuous exploration, continue to evolve, and finally get the optimal decision strategy.

**Definition** **1.** *A MDP (Markov decision process) is defined as a tuple M=(S,A,T,R), where*

- *S is the state space, $S\subseteq R^{n}$.*
- *A is the action space, $S\subseteq R^{m}$.*
- *T is a transfer function S×S×A→[0,1]. $T(s_{t+1}s_{t},a{}_{t})$ is the conditional probability that $s_{t}\in S,a_{t}\in A$ is in state $s_{t+1}$.*
- *R is a reward function R:S×A×S→R, which is a reward obtained by performing operation $a_{t}$ in state $s_{t}$ and transitioning to state $s_{t+1}$.*

Time is defined as discrete t=0,1,2,….$s_{t}$ and $a_{t}$ represent the state and action at time *t*, respectively. The best strategy π=S→A is a strategy for maximizing the expected return:

(2) $$\pi =\arg\max_{\pi }\left(E\left[\sum_{t=0}^{T-1}r(s_{t},a_{t},s_{t+1})\right]\right).$$

*T* is the maximum number of time steps allowed per execution *P* and thus the maximum length of the trajectory. *E* is the expected value after policy p in the equation. The action-value function of the state is defined as

(3) $$Q^{p}\left(s,a\right)=E\left[\sum_{t=0}^{T-1}r\left(s_{t},a_{t},s_{t+1}\right)s_{0}=s,a_{0}=a\right]$$

This is the expected payoff for selecting as an action a strategy *P* in state *s*. $Q^{p}$ is typically used to assess the quality of strategy *P*. $Q^{p}$ and *P* are usually in the form of parametric function approximators for problems with continuous states and action spaces (e.g., automatic driving).

## 4. Framework

**Figure 1:**
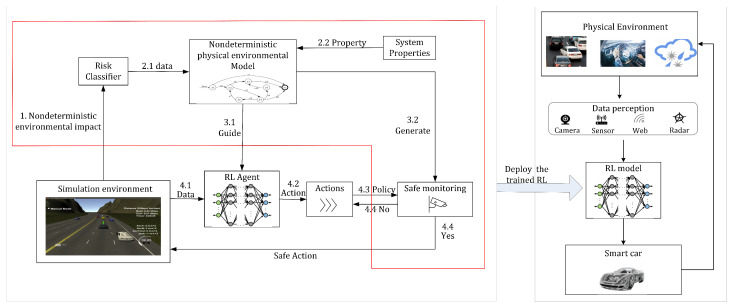
Framework for safe decision controller generation.

The overall proposed framework for the safe decision controller generation proposed in this paper is shown in Figure 1. Figure 1 shows a diagram of the framework generated by the controller which describes the forward flow of data. More specifically, Figure 1 is divided into two components. The left and right regions are processes in the training and physical domains, respectively, and the right region is a process in the physical domain. The controller obtained through the training domain is able to act on the entity.

In the training domain, in the first step, a simulation environment is obtained by accurately modeling the real environment of the autonomous driving system, from which nondeterministic environmental factors are obtained. In the second step, a nondeterministic environment model is constructed by analyzing the classifiers of risk factors and safety properties, and the environment model is defined based on a formal approach to the constraints. In the third step, the environment model is constructed as both a reward machine and a monitor, respectively. The reward machine is used to guide the reward function in RL. The monitor is used to set the monitoring barrier, which monitors the output actions. If the safety properties are not satisfied, the action that satisfies the safety properties are selected from the action set as a way to ensure the decision. The fourth step is the routine execution of reinforcement learning. Data are fed to the agent from the simulation environment, after which an action is obtained for feedback.

## 5. Modeling of the Nondeterministic Physical Environment

There are three types of nondeterministic effects on autonomous driving systems: internal uncertainty, external uncertainty, and sensor uncertainty [32]. Internal uncertainty refers to the differences among the system’s actions, external uncertainty refers to the uncertainty of the system’s surrounding factors, and the sensor uncertainty refers to the deviation of the information received by the sensor. Because the internal and sensor uncertainties are mainly affected by their own design, we mainly focused on the external nondeterminism; that is, the nondeterminism of environmental factors around the system.

Weather and location type are examples of nondeterministic surrounding environmental factors. Temperature, humidity, rainfall, and other factors all have an impact on the weather category. The location category refers to the unpredictability of the activity trajectory of the surrounding objects. The vehicle lane-changing decision-making process is often affected by the nondeterministic environment, in which the interaction with other vehicles is a factor. In the overtaking lane-change scenario, we may be concerned about the vehicle behind us colliding with us because we do not know whether it will accelerate or decelerate. The nondeterministic environment around the vehicle is defined as follows.

**Definition** **2.** *The nondeterministic physical environment model ENV is represented as the tuple ENV=(ER,PR), where*

- *ER is a formal description of the static structure of the nondeterministic physical environment model;*
- *PR is a dynamic description of the nondeterministic physical environment model.*

**Definition** **3.** *The static structure of the nondeterministic physical environment model ER is a four-tuple ER=(Objects,Parameters,Function,Type), where*

- *Objects represents the set of surrounding nondeterministic objects;*
- *Parameters represents the parameter set of nondeterministic factors;*
- *Function represents the probability formula for calculating the category under the nondeterministic environment;*
- *Type represents the set of types of nondeterministic environments.*

**Definition** **4.** *The dynamic description of the nondeterministic physical environment model PR is represented as a six-tuple $PR=(Q,Q_{0},acts,prob,E,L)$, where*

- *Q represents a finite state set;*
- *$Q_{0}$ represents the initial state;*
- *acts is a finite set of actions;*
- *prob: Q×prob→E; prob is the probability of possibility transfer, expressed in a nondeterministic environment;*
- *E: E⊂Q×acts×L×Q is the transfer function;*
- *L: the migration condition.*

The driving style of surrounding vehicles, as a typical factor of the nondeterministic environment, has great influence on automatic driving. Thus, we took the driving style of surrounding vehicles as an example to demonstrate the nondeterministic physical environment model. We assigned a trust level to the surrounding vehicles and attached a value. For example, in the interval [0, 1], if we really do not trust it, its value is 0; if we completely trust it, its value is 1. Typically, we will have an intermediate value depending on how much we know about the vehicle behind us. If we have elements that help the vehicle to perform safe driving (e.g., the amount of speed change, distance between cars, etc.), we can set a value closer to 1, while in the opposite case it is closer to 0. With this level of trust, we can consider the assertion’s veracity.

**Figure 2:**
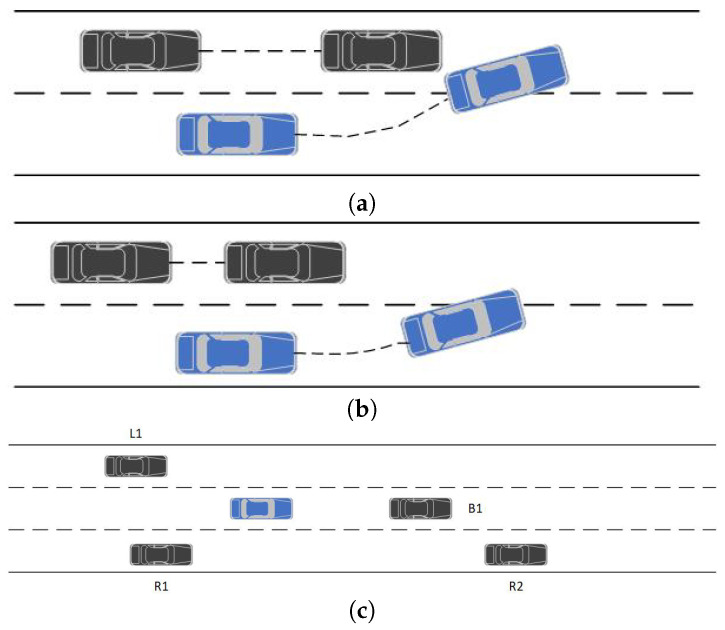
Driving environment. (**a**) Radical collision. (**b**) Conservative successful lane change. (**c**) Surrounding objects.

Figure 2a shows a forced lane change for the rear car’s aggressive style may result in a collision. It is easy to avoid a collision with a conservative style, as shown in Figure 2b. Driving styles were classified as aggressive, normal, or conservative. The aggressive type of rear car should avoid changing lanes, the ordinary type could be taken to change lanes or not, and the conservative type could safely change lanes.

We used the environmental model ER to identify environmental factors and risk probabilities. $Objects\;=\;\{\;\;\;L_{1},B_{1},R_{1},R_{2}\}$ represented the other vehicles in each of the three lanes. $Parameter=\{x_{1},x_{2},x_{3},x_{4},x_{5},x_{6},x_{7}\}$ represented the parameter for calculating the probability. $Function=\{Funp\left(x_{i}|1<i<7\right)\to \left(type,p\right)\}$ represented the formula for calculating the probability. $type\;=\;\{type_{1},type_{2},type_{3}\}$ represented three different driving styles. Each style of transition had a migration relation with probability *p*. We used the parameters in Table 1 to calculate the likelihood of each driving style being judged.

We calculated driving style probabilities using the following formula based on the mean values of the plain Bayesian classifier’s driving style classification parameters [33].

(4) $$Funp=\sum_{i=1}^{7}w_{i}\times B_{xi}$$

**Figure 3:**
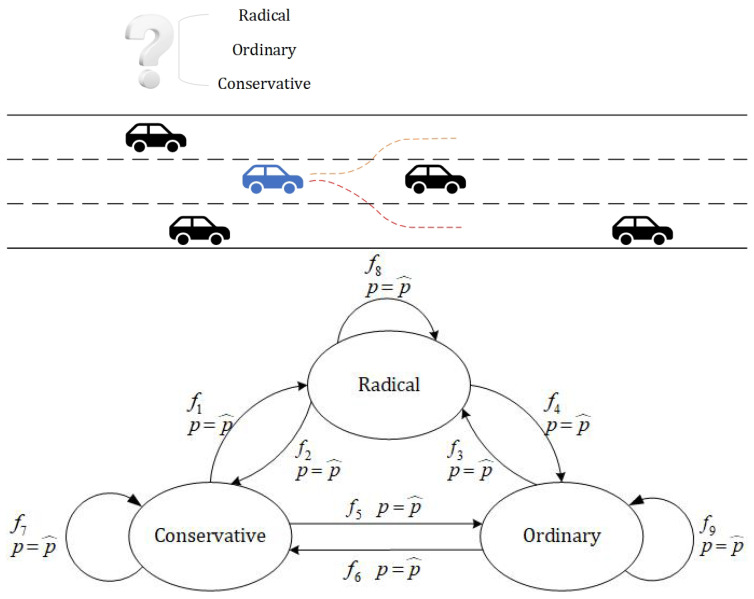
Obstacle avoidance and lane change decision.

$w_{i}$ denotes the weights obtained by training the classifier, where $\sum_{i=1}^{7}w_{i}=1$. $B_{x_{i}}$ denotes whether $x_{i}$ satisfies the computed metric, with a value of 0 or 1. The driving style was predicted by inferring high-level information from low-level data, namely the experimental calculated value and the parameter index. Figure 3 is an example of a dynamic structural representation of a risk model, containing aggressive, conservative, and ordinary types, which were transformed between them by an identification function, and entering a state triggered a reset condition that reset the probabilities.

Self-driving cars on the road need to avoid obstacles as a case of extreme safety. When the blue car on the road overtakes, it has two options: change lane from the left or right. However, the car’s CPS system can only observe the distance between the vehicles and cannot determine the trajectory of the side vehicle. If the rear vehicle is aggressive, it is likely to change lane when the rear vehicle speeds up, resulting in a collision.

## 6. Decision Controller Generation Algorithm for Safety Protocol Guidance

Although RL has been shown to be applicable to complex environmental-interaction-like problems, it faces the problem of lack of interpretability in safety-sensitive domains, and it is difficult for the output policies to guarantee their safety reliability, which poses safety hazards. The reward function of RL is designed to be interpretable in this section. The structure of the reward machine is designed through migration changes between environmental risk model states to guide the setting of the reward function in RL. Additionally, the monitor is designed to guarantee that the output decisions are safe according to the safety properties.

### 6.1. System Specification Modeling

In the automatic driving environment, the safety property that needs to be monitored is often related to time. For example, before changing lanes, it is necessary to turn on the turn signal for 3 s before completing the lane-changing task. This kind of system-safety property with a constraint relationship is usually called a real-time property. The difference between real-time and conventional nature is often reflected in timeliness, which affects the effective time of the decision-making and determines whether the decision can be completed within the specified time.

Because real-time monitoring is time-constrained, it is different from a traditional monitoring process. To monitor the real-time property at run-time, a real-time sequential logic is first used to describe it. In this paper, MTL was used to describe the safety property.

R1: the autonomous vehicle maintains the maximum safe speed of 50 km/h.

$$G(V_{\max}\leq 50)$$

R2: when the vehicleis in the leftmost lane, it is not allowed to change lanes to the left.

G((loc=left)→(¬turnleft_cmd))

R3: when deciding to change lanes to the left, 3 s after the left side still has a safe distance, the lane change can be completed in 1 s.

G(((CMD=turnleft)∧F[0,3]not_left)→F[3,4]turnleft)

R4: the time spent not in the center of the lane while driving shall not exceed 10 s.

G¬(G[0,9](loc≠center))

### 6.2. Setting of Reward Function for Safety Constraint Guidance

The reward function is usually treated as a black box in RL, with no way to understand the settings inside. Because of this approach, the agent-trained decision-maker is unaware of the environmental transfer changes or the state–change relationships and can only obtain the final result. We must accurately perceive the returns from environmental data and allow the agent to learn from environmental interactions. We propose using safety properties and a nondeterministic environment model to guide the setting of the reward function. To express the reward function, we used a reward machine.

**Definition** **5.** *The reward machine RM is a six-tuple $RM=(U,u_{0},acts,\sigma ,E,\tau_{r})$, where*

- *U is a finite set of states;*
- *$u_{0}\in U$ is the initial state;*
- *acts is a collection of actions;*
- *σ are migration conditions;*
- *E is the transfer set, E⊆U×acts×σ×U;*
- *$\tau_{r}$ is a reward function; the current reward $\tau_{ri}=\tau_{r}\left(u_{i-1},u_{i}\right)$.*

The reward machine decomposes the problem of safety constraints into a group of high-level states *U* and defines transitions of $\delta_{u}$ using if conditions. These conditions apply to the safety constraint properties φ, which can be tested to see if they satisfy truth values.

**Definition** **6.** *The reward form RF is a five-tuple: $\tau_{r}=<r,\varphi ,\varphi_{r},risk,risk_{r}>$, where*

- *r represents the base reward set;*
- *φ represents the safety constraint property;*
- *$\varphi_{r}$ represents positive feedback provided by the satisfaction of φ when it is true;*
- *risk represents the risk’s probability of occurrence;*
- *$risk_{r}$ is a function of the negative feedback.*

First, we set basic rewards. The primary concerns are safety and efficiency, which can be obtained by understanding the nature of safety. The system designer wishes to drive the vehicle efficiently while remaining safe on the road in order to avoid collisions. The reward function provides a way to combine prior knowledge and change the decision according to the previous feedback response. One criterion for determining the reward function is to improve the alignment of values between intentions and system goals, as well as to reduce decision errors caused by malicious perturbations of the reward function. Therefore, setting the reward value according to the task result cannot accurately express the advantages and disadvantages of the actual situation, and it needs to be able to measure the different good and bad situations of the execution in different environments.

To improve efficiency, self-driving cars should try to drive as fast as possible while not exceeding the maximum speed limit. Therefore, the following rewards were defined according to the vehicle speed.

(5) $$r_{v}=\lambda_{1}\left(v-v_{res}+\omega_{1}\right)$$

where *v* represents an average speed since the last decision period, $v_{res}$ is the threshold speed limit, $\lambda_{1},\omega_{1}$ are the normalization coefficient, and $r_{v}\in \left[-1,1\right]$ is the reward function of the vehicle speed.

According to the formal description of the safety problem of self-driving cars, in the process of learning the optimal policy, the agent needs to respond to the dynamic evaluation of policy changes in a dynamic environment. Different decisions are made in the current environment due to the different driving styles of other driving vehicles around, resulting in different changes in the distance between vehicles. Due to the different driving styles of other surrounding vehicles and the different decisions made in the current environment, the change of vehicle distance is also different. A big reason for collision accidents on the road is that the vehicle distance from the surrounding vehicles is far less than the safe distance. Therefore, the reward function of dynamic change concerning vehicle distance was formulated to measure whether it was good or bad. According to the critical safety time distance [34], it was defined as

(6) $$d=\frac{{\left(v_{r}+|a_{r}|t_{\tau }-v_{f}\right)}^{2}}{2(|a_{r}|-|a_{f}|)}-\frac{|a_{r}|t_{\tau }^{2}}{2}$$

where $v_{r}$ is the vehicle speed of the rear vehicle, $v_{f}$ is the vehicle speed of the front vehicle, $a_{r}$ is the acceleration of the rear vehicle, $a_{f}$ is the acceleration of the front vehicle, and $t_{\tau }$ is the response lag time. The acceleration *a* and vehicle speed *v* were calculated by the instantaneous displacement, and the formula was as follows:

(7) $$\left\{\begin{array}{c} v=\frac{d(t+\Delta t)-d(t)}{\Delta t}\\ a=\frac{v(t+\Delta t)-v(t)}{\Delta t} \end{array}\right.$$

The violation of the safety distance constraint causes RL to make the vehicle drive at a safe distance during the learning process. According to the relationship of the safety distance, we gave a reward function about the distance:

(8) $$r_{d}=\lambda_{2}\left(d-d_{act}+\omega_{2}\right)$$

where *d* represents the critical safety time distance, $d_{act}$ is the observed distance, $\lambda_{2}$ and $\omega_{2}$ are the normalization coefficients, and the reward function of the vehicle speed is $r_{d}\in \left[-1,1\right]$.

According to the automaton representation of safety property transformation, when facing the leftmost or rightmost side of the road, the vehicle is not allowed to change lanes left or right, so a negative feedback reward $r_{illegal}$ is set. When the decision is made successfully, a positive feedback reward $r_{succeed}$ is given. If a collision occurs, it is a great safety problem, and a strong negative feedback reward $r_{collision}$ is set.

At the same time, there is a possibility that the RL will cause the vehicle to make a lane change even if there is no vehicle in front of it. There is no safety accident following a legal lane change, but this violates one of the constraints (frequent lane changes increase the probability of collision and reduce the number of lane changes to reach the destination as soon as possible). Thus, in order to alter this phenomenon, we established a negative feedback reward $r_{valid}$.

(9) $$r=\left\{\begin{array}{cc} r_{y}+r_{d} & \;\mathrm{Current}\;\mathrm{Lane}\;\\ r_{y}+r_{d}+r_{success} & \;\mathrm{Succeed}\;\mathrm{Change}\;\\ r_{y}+r_{d}+r_{invalid} & \;\mathrm{Invalid}\;\mathrm{Change}\;\\ r_{illegal} & \;\mathrm{Illegal}\;\mathrm{Change}\;\\ r_{collision} & \;\mathrm{Collision}\; \end{array}\right.$$

**Figure 4:**
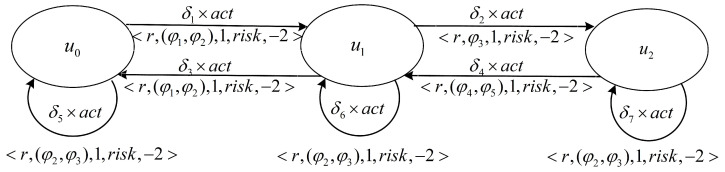
Reward machine.

We designed the rewards directly from high-level specifications to make the reward synthesis problem more formal and easier to interpret and handle. Figure 4 shows a reward machine with reward specification $\tau_{r}=<r,\varphi ,\varphi_{r},risk,risk_{r}>$.

Figure 4 shows the state change of an autonomous vehicle in a three-lane highway. $U=(u_{0},u_{1},u_{2})$ represents a high-level state in the right lane, the middle lane, and the left lane. $\delta =(\delta_{1},\delta_{2},\delta_{3},\delta_{4},\delta_{5},\delta_{6},\delta_{7})$ represents a state transition, which is determined by the previous state and the current state. Where $<r,\left(\varphi_{1},\varphi_{2}\right),\varphi_{r},risk,risk_{r}>$ is the reward function for changing from the right lane state $u_{0}$ to the middle lane $u_{1}$, and *r* is the set basic reward. $\left(\varphi_{1},\varphi_{2}\right)$ denotes the property constraint of changing lanes at a safe distance and whether there is speeding, respectively, $\varphi_{r}$ denotes the positive feedback reward of 1 when the property φ is true. risk is the risk probability of identifying the nondeterministic environment, which is obtained by quantifying the nondeterministic environment risk mentioned above, $risk_{r}$ is −2, and the coefficient of negative feedback given to the probability risk function is −2.

### 6.3. Feedback Based on Safety Monitoring Barrier

**Figure 5:**
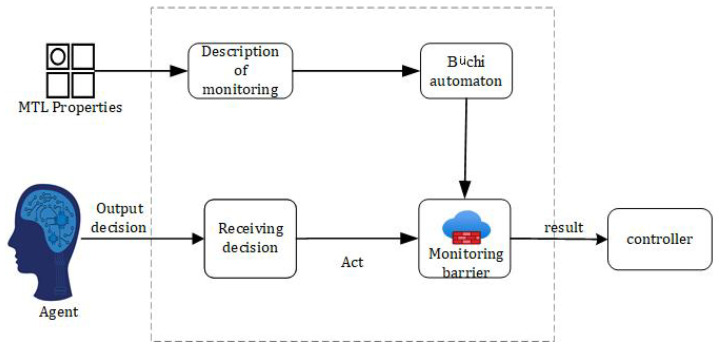
Monitoring barrier.

The ability of a CPS real-time system to react to surrounding data in a timely and accurate manner and then hand it over to the controller for response is critical to safe system operation. It is also possible to quickly determine whether the system will be impacted in a way that is not expected. To monitor the system’s operation, a runtime monitoring barrier was established. Its safety was verified for various safety properties, i.e., whether the safety constraints were violated and that the controller performed safe operations. Figure 5 depicts the structure of the monitoring barrier.

The monitoring barrier intercepts the intelligence’s decision, accepts the decision action as input, and determines whether the action decision satisfies the safety property constraint. The monitoring barrier has two primary functions: satisfaction verification and safety assurance output.

**Definition** **7.** *Satisfaction verification. When the monitor verifies the satisfaction of a property, it can be considered as a Boolean function output to determine whether the current system state satisfies the safety property constraint through the state migration triggered by the selected action. It is expressed as*

(s,act,φ)→bool

*where s∈S is the current state, act∈acts is the selected action, φ is the safety property specification, and the output is {true,false}.*

**Definition** **8.** *Safety assurance output. When the input action does not satisfy the property statute, a safe new action should be selected from the set of candidate actions.*

### 6.4. Generation Algorithm of Safe Decision Controller

In this section, monitoring barriers transformed by safety properties are used in conjunction with the DRL algorithm to create a controller capable of making safe decisions through training. We propose a general safety learning algorithm, which sets the reward function guided by the safety property, and then uses the monitoring barrier to verify the output of the algorithm, so as to ensure the safety of the output decision and to be able to apply the safety RL algorithm to the safety-critical CPS system. By adding a monitor to the DRL algorithm as the intelligence’s supervisory layer, the information exchange between the environment and the intelligence is constantly monitored, and the decision’s safety is judged through verification, so that when a risk is perceived, a safe decision can be provided, and the risk can be avoided as much as possible.

Algorithm 1 gives the algorithm for solving the optimal action for the conventional DQN. In the traditional DQN algorithm, the agent selects an action with probability p, then obtains immediate returns and subsequent states from the environment feedback, and finally performs the update function.
**Algorithm 1 **DQN Algorithm**Input:** 
MDP=(S,A,T,R),done,update **Output:** Optimal policy π
  1: Init (π)  2: $s_{t}\leftarrow s_{0}$  3:
a←NOP  4: **while** 
$s_{t}!=done\;\mathrm{and}\;episode<episodes$ 
**do**  5:    // Iterative selection of optimal value  6:    $A^{{}^{\prime }}=A$  7:    $a\leftarrow Random_{S}elect\left(A^{{}^{\prime }}\right)$  8:    $update(s_{t},a,\pi )$ // Update parameters  9:    Go to the next state $s_{t+1}$ 10:** end while**


However, when faced with inexperienced scenarios and complex decision-making tasks, black-box-based DRL systems do not guarantee the safety of the system and the complex interpretability of the reward function settings for complex tasks, so using the traditional DQN algorithm to generate decisions does not work very well. In the following, the nondeterministic environment model is combined into the algorithm implementation based on the methodology from the previous sections.

Algorithm 2 shows the algorithm for safe decision-making generation. When the environment is accurately modeled, the system selects from a set of validated safety actions; otherwise, the system selects conservative policy actions from the action space. The inputs are a reinforcement model MDP=(S,A,T,R), a reward machine $\tau_{r}\;=$$<r,\varphi ,\varphi_{r},risk,risk_{r}>$, an end state, and an update of a function that records state transitions. The monitoring barrier is used to monitor the system for previously observed states, previous control actions, and the current state to satisfy safety requirements. The controller receives the decision whenever the monitoring barrier is true.
**Algorithm 2 **Generation algorithm for the safe decision controller**Input:**$MDP=\left(S,A,T,R\right),\tau_{r}=\;<r,\varphi ,\varphi_{r},risk,risk_{r}>,done,update$ **Output:** Optimal safety policy π  1: Init (π)  2: $s_{t}\leftarrow s_{0}$  3: a←NOP  4: **while** $s_{t}!=done\;\mathrm{and}\;episode<episodes$ 
**do**  5:    // Iterative selection of optimal value  6:     $A^{{}^{\prime }}=A$  7:    $a\leftarrow choose(A^{{}^{\prime }})$ // Select action  8:    env←Environmentalsampling  9:    **while** $monitor(s_{t},a,env)$ **do** // monitor 10:        $A^{{}^{\prime }}\leftarrow A^{{}^{\prime }}-a$ 11:        **if** $A^{{}^{\prime }}\;!=\;⌀$ **then** 12:           $a\leftarrow choose(A^{{}^{\prime }})$ 13:        **else** 14:           $a\leftarrow choose(a_{\mathrm{conservative}}\in A)$ 15:           break 16:        **end if** 17:    **end while** 18:    $update(s_{t},a,\pi )$ // Update parameters 19:    Go to the next state $s_{t+1}$ 20: **end while**


Lines 1–3 show the process starting in its initial state. Lines 4–20 represent the choice of the next action and its execution until the termination state is reached or the maximum number of rounds is reached. Lines 6–7 describe the chosen action. Line 8 describes the process of acquiring environmental parameters. Lines 9–17 express the monitoring barrier, which monitors the state, environment, and the selected action; if it returns true, an action is output; otherwise, the greedy idea is used to choose another action from the action subset; and if none of them return true, a conservative policy action is output. Line 18 updates the state based on the environment and the learning model. The entire algorithm consists of two layers of loops, the first of which is about the abort state and the number of training rounds (episodes). The second loop is a monitor that selects the optimal values from the action space for the output. The algorithm complexity is $O(n^{2})$.

## 7. Evaluation

### 7.1. Training Details

**Figure 6:**
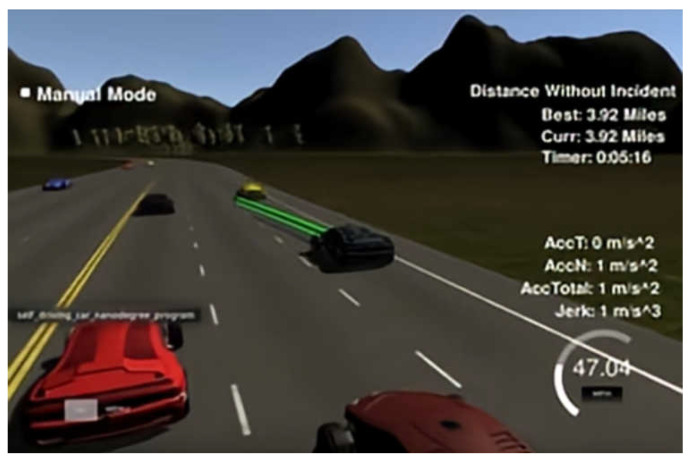
Simulation environment.

In our study, we used the path-planning project simulator from the Udacity self-driving car project [35]. The simulator can provide the vehicle’s positioning and sensor fusion data, obtain the map information around the vehicle, give a visual graphical interface, and produce an effective definition and simulation of time and position. It also provides a visual interface and a controller module, which can communicate information with each other through sockets. The observation-state information data in the simulation environment was transmitted to the algorithm, and then the high-level action decisions calculated by the algorithm were transmitted back to the target vehicle agent. The simulator is shown in Figure 6.

The experimental scenario was designed as a self-driving car traveling at 50 MPH on a highway with surrounding vehicles traveling at 40–60 MPH. The car should drive as close to the speed limit as possible. When facing a blocked road section, it should try to change lanes to overtake. At the same time, the vehicle should ensure safety when making lane-change decisions. The experimental environment we used is shown in Table 2. As for the safety properties, we chose a safe distance for side vehicles when changing lanes. The property is Section 6.1—R3.

The simulation environment was a three-lane highway road map. A 45 × 3 matrix was used as the road’s state-information representation, with three columns representing the corresponding three lanes and each vehicle in each lane occupying four cells. The number filled in the matrix was the speed of the vehicle. The action space was chosen as $a=a_{0},a_{1},a_{2}$, $a_{0}$ for keeping the current lane, with $a_{1}$ for changing lanes to the left and $a_{2}$ for changing lanes to the right. The reward function was defined by the reward machine in Section 6.

The algorithm used in this paper was based on Tensorflow’s DQN algorithm [36]. We improved the safe DQN algorithm by incorporating safety monitoring to implement the car intelligent lane-change decision. In our study, we trained the two algorithms for 100 rounds of deep reinforcement, with each round consisting of a loop on a 6946-meter-long highway, which took about 6 min to complete. We set all the parameter weights of nondeterministic probabilities to 1/7. During the training process, after each round of the task was completed, the vehicle agent returned to the initial point for the next round of training.

### 7.2. Comparing with Tensorflow’s DQN Algorithm

We compared the average speed, lane-change time, and collision time for each phase of the two methods.

**Figure 7:**
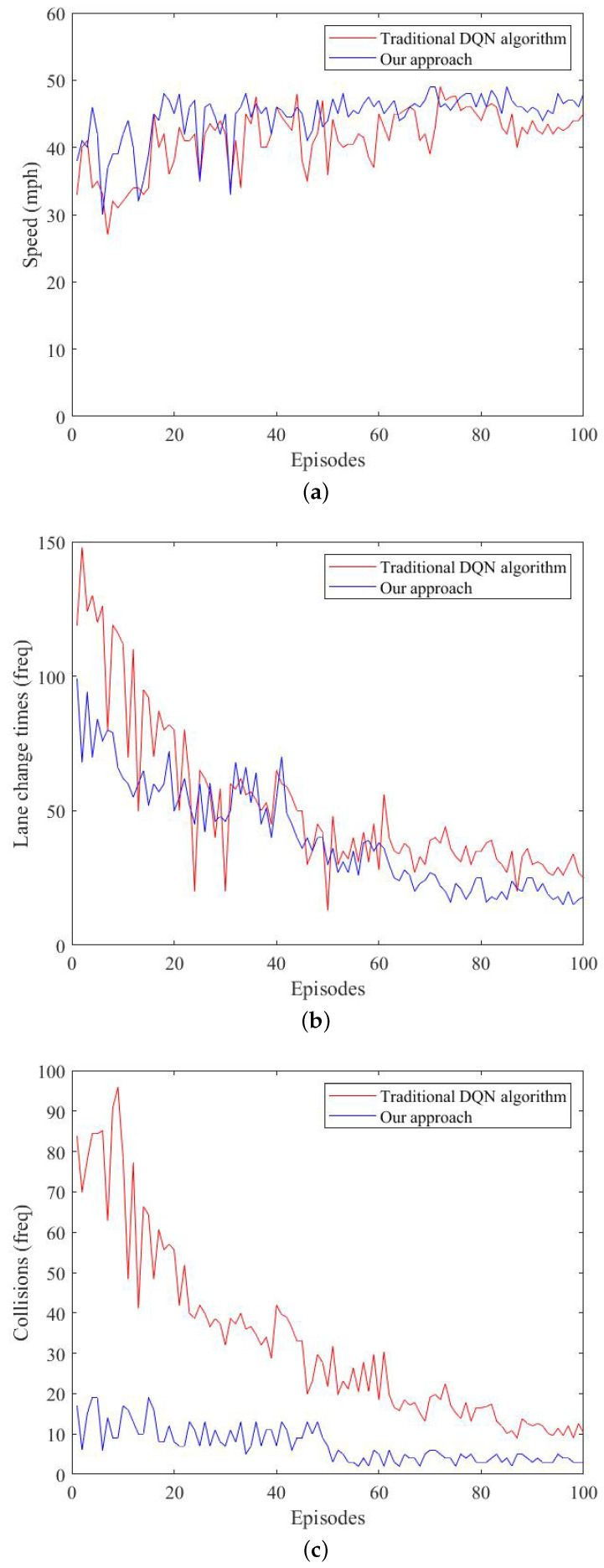
Comparison of traditional and improved methods. (**a**) Comparing speed. (**b**) Comparing lane-change times. (**c**) Comparing collisions.

Figure 7 shows that the car’s behavior was ideal. Figure 7a shows the average speed obtained by the algorithm in each round of training. As the number of rounds increased, the average speed was closer to the speed threshold, and the vehicle agent could complete the route task more efficiently. The vehicle’s speed was greatly improved in the 20th round of training. At the same time, when the training round reached 72 rounds, the safety RL algorithm could achieve speed stability faster; Figure 7b shows the number of lane-changing events in each round. The frequency of lane changing was significantly reduced, and the average vehicle speed was increased. A faster speed was obtained by fewer lane-changing behaviors, and a faster speed in the task was met to reach the destination. Figure 7c shows the number of collisions per round. The number of intelligent vehicle collisions decreased with the increase in the number of training rounds, which indicated that the intelligent vehicle learned correct driving actions through exploration and RL, reducing the number of collisions.

The graph shows a counterexample at rounds 30–40, when the algorithm had not yet stabilized, which produced a high collision rate. The figure compares the traditional way of designing the DQN algorithm with our method over the course of 100 training rounds. The overall average increase in driving speed was 3.371 MPH, the overall average reduction in the number of lane changes was 11.64, and the overall average reduction in the number of collisions was 25.52. Collisions could be avoided as much as possible by using the safety RL algorithm; however, the risk of collision still existed in the experiment, which we observed through the visual interface when the distance between the vehicle and the rear vehicle was too small when changing lanes, causing a collision when decelerating. From our experimental results, we observed that the use of safety monitoring in RL could make the agent’s decision-making safer while ensuring the algorithm’s decision-making output converged faster.

We compared the trained model with several different approaches, including a random lane-change strategy with rule constraints, a rule-based lane-change strategy, and a pure DQN lane-change strategy. Compared with several other methods, the car’s average execution speed in our method was slightly better. Additionally, our method had a higher security rate than several of the remaining policies, which ensured the safety of the decision. This showed that our approach achieved a more efficient and safer performance compared with other methods (see Table 3).

### 7.3. Assessing the Effect of the Nondeterministic Environment

**Figure 8:**
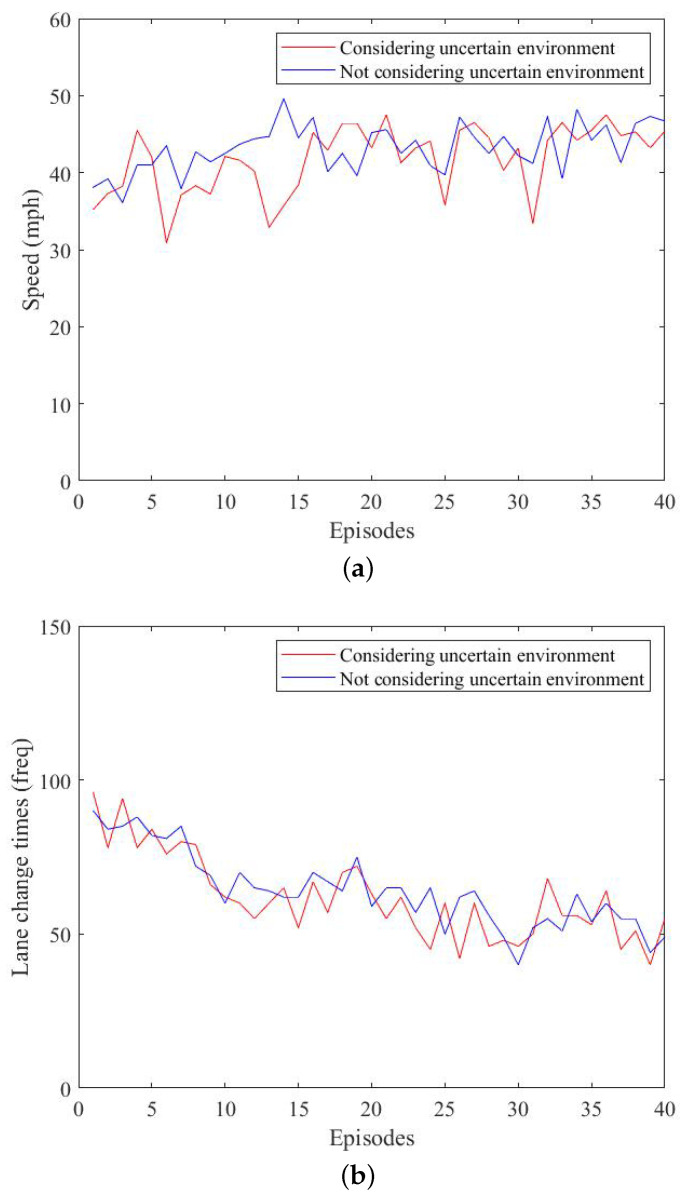

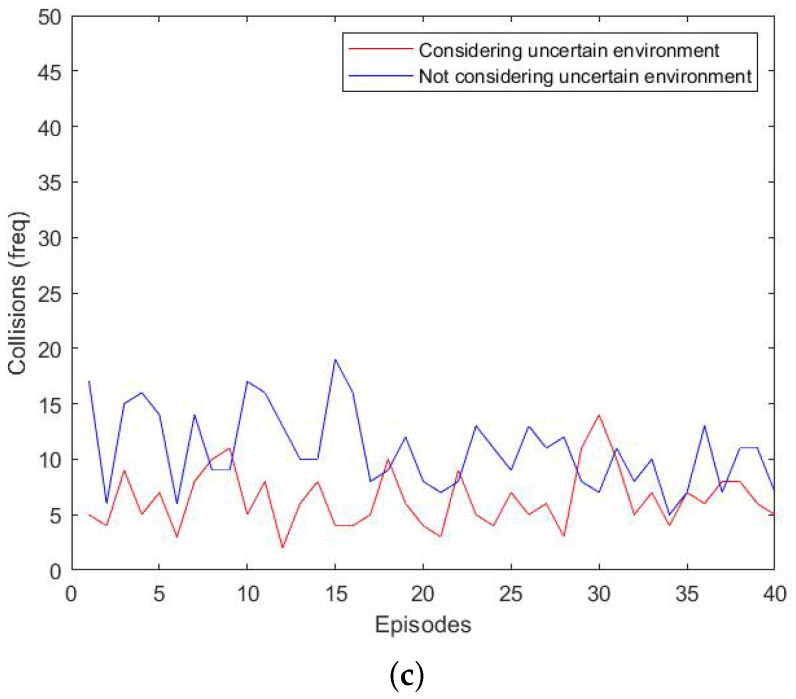
Comparison of nondeterministic environmental impact. (**a**) Comparison of speed under environmental factors. (**b**) Comparison of lane change times under environmental factors. (**c**) Comparison of collisions under environmental factors.

Figure 8 shows a comparison experiment between the monitor considering the effect of the nondeterministic environment and the case without it. We intercepted the data from rounds 0–40. In the figure, we compared the effect of considering environmental factors with not considering environmental factors and analyzed the first 40 rounds with significant differences. Our method showed a significant reduction in the number of collisions; however, at the same time, the driving speed was slightly lower compared with the conventional method. This is because our method considered the uncertain driving style variation, and if there was a high probability of risk, it chose to follow the car, resulting in a lower speed.

Through the above analysis, our method overcame the disadvantage of not being able to guarantee safety in the nondeterministic environment compared with the traditional method and achieved the safety requirements of autonomous driving.

## 8. Conclusions

In our study, we proposed a DRL-based generation method for autonomous driving CPS decision controllers to address the problem of obtaining a safe decision controller for CPS systems in nondeterministic environments. First, we obtained a simulation environment by accurately modeling the real environment of the autonomous driving system, from which we obtained nondeterministic environmental factors. In the second step, we constructed a nondeterministic environment model by analyzing the classifiers of risk factors and safety properties, and then we defined the environment model based on a formal approach of the constraints. In the third step, we constructed the environment model as both a reward machine and a monitor. We used the reward machine to guide the reward function in RL and utilized the monitor to set the monitoring barrier, which monitored the output actions. If the safety properties were not satisfied, we selected the action that satisfied the safety properties from the action set to ensure the decision. The fourth step was the routine execution of reinforcement learning. Data were fed to the agent from the simulation environment, after which an action was obtained for feedback. Through experiments, we verified the feasibility of our method, and the addition of our method ensured the safety of the decisions. We verified the practicability of formal modeling and verification methods in nondeterministic environments and strengthened the learning process from safety property specification and formal modeling constraints. The definition of a reward function in a practical application can be explained by the formal method. Our method provides an idea for the combination of formal methods and reinforcement learning.

However, our work was carried out in a three-lane motorway environment, which still lacks validation for more complex scenarios. In future work, we also need to consider optimizing the design and training of reinforcement learning safety constraints, considering the influence of traffic rules. Secondly, we can also improve the reward–reward function of the reinforcement learning by making more precise definitions to improve the efficiency of learning and the interpretability of learning strategies. Finally, simulations should be validated with more nondeterministic environmental factors to gradually promote the diffusion and practical application of safety-based reinforcement learning in unmanned systems.

## Figures and Tables

**Table 1:** Various characteristics of the parameters.

	Parameters’ Characteristics
$x_{1}$	Average velocity
$x_{2}$	Standard deviation of vehicle speed
$x_{3}$	Maximum lateral acceleration
$x_{4}$	Standard deviation of lateral acceleration
$x_{5}$	Following distance
$x_{6}$	Lane change time
$x_{7}$	Lane change clearance

**Table 2:** Experimental settings for the simulation.

Experimental Settings
Processor: Intel Xeon W-2225
Video card: NVIDIA Quadro rtx5000, 16 GB
Memory: 128 GB
Operating system: Ubuntu 18.04
Development language: Python 3.6
Development framework: tensorflow 2.4.0

**Table 3:** Advantages and disadvantages of different methods.

Method	Avg Speed	Avg Lane Changes	Safety Rate
Random action [37]	44.59	152.60	0.6
Rule-based [38]	45.22	8.40	0.6
DQN-based [39]	46.16	37.40	0.2
Our method	46.73	11.80	0.9

## Data Availability

Not applicable.
